# A Printable and Conductive Yield-Stress Fluid as an Ultrastretchable Transparent Conductor

**DOI:** 10.34133/2021/9874939

**Published:** 2021-12-14

**Authors:** Qianying Lu, Yunlei Zhou, Xiangfei Yin, Shitai Cao, Xiaoliang Wang, Desheng Kong

**Affiliations:** ^1^College of Engineering and Applied Sciences, State Key Laboratory of Analytical Chemistry for Life Science, and Jiangsu Key Laboratory of Artificial Functional Materials, Nanjing University, Nanjing 210046, China; ^2^Key Laboratory of High Performance Polymer Materials and Technology of Ministry of Education, Department of Polymer Science and Engineering, School of Chemistry and Chemical Engineering, Nanjing University, Nanjing 210046, China

## Abstract

In contrast to ionically conductive liquids and gels, a new type of yield-stress fluid featuring reversible transitions between solid and liquid states is introduced in this study as a printable, ultrastretchable, and transparent conductor. The fluid is formulated by dispersing silica nanoparticles into the concentrated aqueous electrolyte. The *as*-printed features show solid-state appearances to allow facile encapsulation with elastomers. The transition into liquid-like behavior upon tensile deformations is the enabler for ultrahigh stretchability up to the fracture strain of the elastomer. Successful integrations of yield-stress fluid electrodes in highly stretchable strain sensors and light-emitting devices illustrate the practical suitability. The yield-stress fluid represents an attractive building block for stretchable electronic devices and systems in terms of giant deformability, high ionic conductivity, excellent optical transmittance, and compatibility with various elastomers.

## 1. Introduction

The rapid proliferation and evolution of consumer electronics have stimulated the growth of stretchable electronic technology featuring compliant mechanical properties [1–3]. Stretchable devices are able to be bent, twisted, stretched, and interfaced with moving objects, which are readily integrated into various emerging fields including health monitoring systems [4–6], advanced prosthetics [7–9], and human-machine interfaces [10, 11]. Among various building materials, the stretchable transparent conductor is a key enabler for deformable forms of optoelectronic devices such as light-emitting devices [12–16] and photodetectors [17]. The prototypical forms of stretchable transparent electrodes are electronic conductors of sliver nanowires randomly assembled over elastomer substrates to form percolation conductive networks [12, 18]. The degradations of optoelectronic performances upon tensile deformations are ascribed to the sliding between silver nanowires and damages to internanowire junctions [19, 20]. Sliver nanowires with high area loading and plenty of junctions are often employed to achieve improved stretchability at the price of reduced optical transmittance [19–21]. In addition, the conductive properties of these electrodes typically show rapid deteriorations under biaxial tensions unless carefully engineered microstructures are employed [22, 23].

Although transparent electronic conductors struggle to achieve large deformability, the ionic counterparts have already demonstrated the suitability for the application settings of stretchable electronics. Liquid-state ionic conductors, such as ionic liquids and electrolyte solutions, embedded in elastomers represent a class of ionically conductive materials with the ultimate compliance and stretchability [24–26], The device fabrication process typically requires microfabricated molds to confine the readily flowable liquids [27]. Ionically conductive gels, formed by immobilizing liquid state conductors inside crosslinked polymer matrices, show improved processability as solid-state materials, which are compatible with various patterning techniques including photocrosslinking [28, 29], laser cutting [30, 31], and screen printing [32]. The potential fracture of the polymer matrix upon tensile deformations, however, requires substantial efforts to design the network structure for large stretchability [33, 34]. In addition, special surface modifications on elastomer substrates are often necessary to establish a stable and strong interface with these gels for the fabrication of functional devices, in order to prevent interfacial delamination at large tensile strains [35, 36].

A class of non-Newtonian fluids known as yield-stress fluids represents attractive soft materials seamlessly combining the attributes of solids and liquids [37], which are the enabler for a broad range of applications including food products [38, 39], three-dimensional printings [40–42], and drug delivery systems [43, 44]. These materials maintain the shape with solid-state appearances at static conditions and readily flow when the applied stress exceeds a critical value. A rich variety of chemistries and structural designs are available to formulate functional, including colloid suspensions, emulsions, entangled polymer liquids, and electro/magnetorheological fluids [45, 46]. The fully reversible solid-to-liquid transitions of yield-stress fluids allow large deformations by flowing in liquid states and thorough recovery of solid properties irrespective of the strain history [37, 45]. These desirable characteristics have rarely been harnessed in stretchable electronic devices.

In this study, we introduce a highly conductive yield-stress fluid as the printable ink for stretchable transparent conductors. The rheological complex fluid consists of colloid silica nanoparticle suspension in highly concentrated aqueous electrolyte, which is compatible with the screen printing technique for the deposition of delicate and arbitrary features over various elastomers. The *as*-printed features exhibit a solid appearance to enable subsequent encapsulation in elastomers. The transition into liquid-like behavior of yield-stress fluid upon tensile deformation results in conformal interactions with the elastomeric matrix and ultrahigh stretchability up to the fracture strain of the elastomer. The successful implementation of these transparent electrodes in wearable strain sensors and stretchable light-emitting devices demonstrates the practical suitability. The yield-stress fluids reported here features giant stretchability, high ionic conductivity, excellent transparency, exceptional durability, and facile integration with elastomeric structures, which represents an enticing building block for deformable optoelectronic devices and systems.

## 2. Results

**Figure 1:**
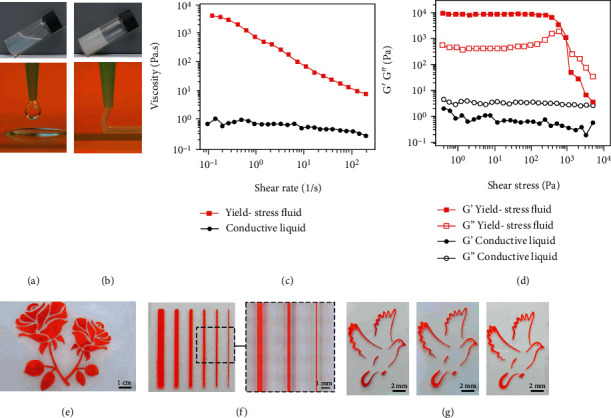
Formulation of yield-stress fluid as printable ink. (a) Ionically conductive liquid loaded in a vial (top) and squeezed through a needle (bottom). (b) Yield-stress fluid loaded in a vial (top) and extruded through a needle (bottom). (c) Viscosity versus shear rate for the conductive liquid and the yield-stress fluid. (d) Storage moduli (*G*′) and loss moduli (*G*^″^) as a function of shear stress. (e) Optical image of a presentative rose-shaped pattern printed on a TPU substrate. (f) Optical and optical microscope images of an array of line-shaped features. (g) Optical images of pigeon-shaped patterns on various elastomer substrates including SIS (left), silicone (middle), and PVDF-HFP (right).

An ionically conductive liquid is prepared by dissolving high concentration LiCl salt in an aqueous PEO polymer solution, which generates droplets by squeezing it out from a fine needle (see Figure 1(a)). A yield-stress fluid is formulated by further dispersing hydrophilic fumed silica into the conductive liquid and behaves like a solid-state gel under static conditions. In the extrusion process from a needle, the yield-stress fluid transforms into the flowable state and thereby forms a continuous filament (Figure 1(b)). The reversible transitions between liquid and solid states are further revealed by manipulating the fluid in static and shaking conditions, as shown in Movie S1. The solid behavior of the yield-stress fluid under stationary conditions is associated with the formation of a three-dimensional network of chain-type silica aggregates [47]. The network is easily disrupted under the shear stress and rapidly recovered when the external loading is removed [48]. Notice that the PEO effectively enhances the stability of the yield-stress fluid to suppress the potential sedimentation during long-term storage (see Figure S1). The rheological characteristics are systematically characterized by a Hakke RheoStress 600 rheometer, as shown in Figures 1(c) and 1(d). The conductive liquid is essentially a Newtonian fluid with a low viscosity (*η*) of ~1 Pa·s almost independent of the shear rate. The liquid characteristic is confirmed by the fact that the storage modulus (*G*′) is lower than the loss modulus (*G*^″^) over the entire range of the applied shear stress (see Figure 1(d)). In contrast, the addition of fumed silica effectively increases the viscosity to 4 × 10^3^ Pa · s at the quasi-static condition of 0.1 s^−1^. The viscosity shows a strong shear-thinning behavior with rapid reduction by over 2 orders of magnitude by increasing the shear rate from 0.1 to 200 s^−1^. The ink also exhibits a plateau value of *G*′ ~ 8.9 × 10^3^ Pa, which is more than 15 times the value of *G*^″^ to suggest a solid-like behavior in stationary conditions. The moduli curves have a crossover point of the shear yield stress (*τ_y_*) at 900 Pa, corresponding to the reversible transition between solid and liquid states. The rheological properties of the fluids containing varying concentrations of fumed silica are further measured and shown in Figure S2. The additional silica increases viscosity, quasistatic storage moduli, and shear yield stress by forming dense network structures. In addition, the combined rheological modifiers of PEO and fume silica synergistically enhance the storage modulus and the shear yield stress (see Figure S3). In the yield-stress fluid, the polymer chains of PEO are physically absorbed onto silica surfaces and thereby strengthen the interparticle interactions of the three-dimensional colloid network [49, 50].

The yield-stress fluid is well suited as the ink for screen printing that behaves like a low-viscosity liquid under high shear when pressed through the stencil openings, followed by rapid transformation back into solid structures to prevent spreading in the absence of additional shear [51–54]. As illustrated in Figures 1(e), a representative rose-shaped pattern is printed onto a TPU substrate exhibiting intact morphology and smooth edges. Notice that the fluid is colored in red with ponceau 4R dye to facilitate visual identifications. In Figure 1(f), an array of line-shaped patterns is created to reveal the minimum feature dimension as ~200 *μ*m, thereby demonstrating the suitability for printing delicate patterns. The rheological characteristics of the yield-stress fluid largely dictate the printing quality. The partially collapsed feature is observed with fluid formulations containing an insufficient amount of fumed silica (see Figure S4). The excellent replication of the stencil mask requires large viscosity and storage moduli with a high concentration of silica modifier to suppress the lateral spreading of the printed features (see Figure S4 and Figure S5). In addition to TPU, the optimized yield-stress fluid is compatible with a variety of stretchable substrates, including SIS, silicone, and PVDF-HFP, as shown in Figure 1(g).

**Figure 2:**
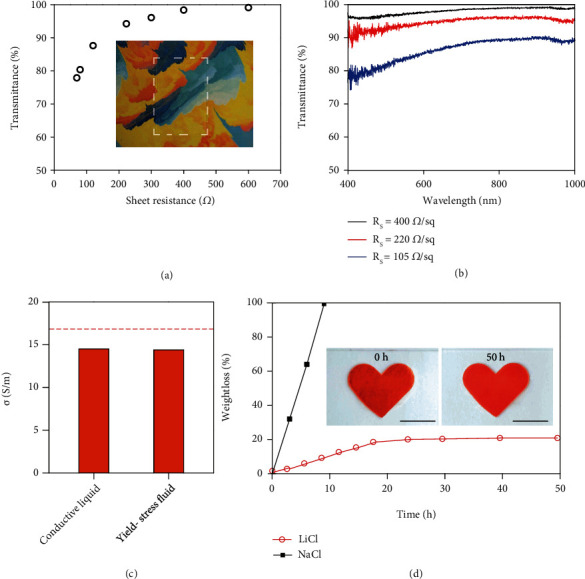
Yield-stress fluid electrodes as transparent conductors. (a) Transmittance spectra of electrodes with different sheet resistance in the wavelength range from 400 to 1000 nm. Inset: Image of a representative transparent electrode with a sheet resistance of 400 *Ω*. (b) Optical transmittance (at 550 nm) versus sheet resistance for electrodes based on the yield-stress fluid. (c) Conductivity of the conductive liquid and the yield-stress fluid. The conductivity of the original 10 M LiCl aqueous solution is marked by the dotted line for comparison. (d) Water loss as a function of storage time in an environmental chamber at 23°C and 20% relative humidity for yield-stress fluids based on LiCl and NaCl. Inset: Image of a LiCl-based electrode acquired at 0 and 50 h. Scale bar: 1 cm.

As revealed by the inset of Figure 2(a), the printed yield-stress fluid electrode is highly transparent primarily due to the high water content (>85 w/w %). Figure 2(a) shows optical transmittance as a function of sheet resistance controlled by adjusting the thickness of the electrode. The yield-stress fluid electrodes demonstrate competitive optoelectronic properties in terms of Rs = 400 *Ω*/sq. at *T* = 98.4%, Rs = 220 *Ω*/sq. at *T* = 94.2%, and Rs = 90 *Ω*/sq. at *T* = 83.5%, respectively. In Figure 2(b), the fairly flat transmittance spectra suggest color-neutral characteristics, which essentially eliminate chromatic corrections in device applications [55]. The attractive performances of these electrodes are largely associated with the exceptional conductivity of 14.4 S/m. In Figure 2(c), 10 M LiCl aqueous solution has abundant dissociated ions and a high conductivity of 16.2 S/m, which represents the upper limit for its derivatives. The addition of 5 w/v % PEO gives rise to ~10% decrease in conductivity as compared with that of the pristine aqueous electrolyte. The conductive liquid of the polymer solution is a Newtonian fluid with increased viscosity to hinder the ionic diffusions [56, 57]. The subsequent incorporation of 10 w/v % fumed silica gives rise to negligible changes in conductivity, because the colloid network is phase-separated from the electrolyte with limited influences on the ion transports [58]. In practice, the concentration of fumed silica is systematically modulated to achieve the optimal printing quality. The PEO is kept in the low concentration range due to the negative impact on the ion conductivity (Figure S6). Accordingly, the optimized yield-stress fluid functions as a high-performance ionic conductor by inheriting the excellent conductivity of the original aqueous electrolyte.

The water retention capability is a key requirement for stable long-term operations. The water loss is evaluated by monitoring the weight as a function of storage time in a dry environment. The rheological modifiers are ineffective to prevent the yield-stress fluid from drying out, as illustrated in Figure S7. The salt choices are critical for yield-stress fluids to stay hydrated. As shown in Figure 2(d), the water loss of LiCl-based fluid gradually approaches the steady-state value of ~20%. The exceptional water retention is further confirmed by the preservation of the overall morphology of the printed electrode after 50 h storage under dry air conditions (see the inset of Figure 2(d)). In contrast, continuous weight loss through evaporation is observed in NaCl-based fluid. The large differences in water retention are associated with the variations in ionic hydration degrees of the dissolved salts [59]. The highly hydratable LiCl salt is well suited to improve water retention, because the strong bonds between cation/anion-water pairs provide energy barriers that prevent water molecules from evaporation [60]. In addition, the water retention capability is also directly correlated with LiCl concentration, as illustrated in Figure S7. The reduced water loss at high salt concentration is ascribed to the decreased fraction of free water molecules that readily evaporate.

**Figure 3:**
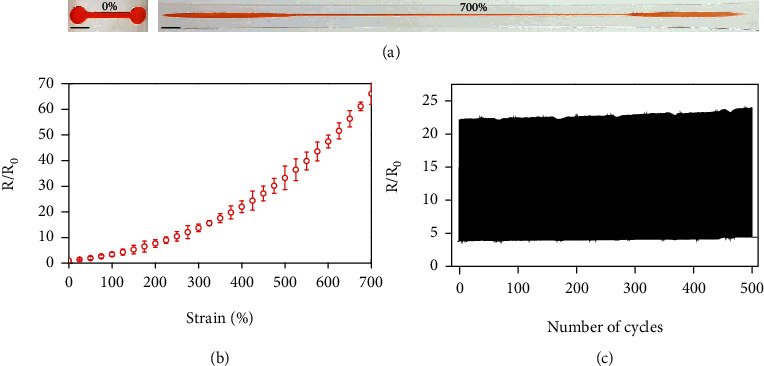
Yield-stress fluid electrode under uniaxial tensile deformations. (a) Optical images of a yield-stress fluid electrode at 0% and 700% strains. Scale bar: 5 mm. (b) Normalized resistance as a function of strain. (c) Evolution of normalized resistance over 500 stretch relaxation cycles in the strain range from 100% to 400%.

The printed yield-stress fluid pattern stays as solid-state structures to enable facile encapsulation by elastomers, which functions as a highly deformable conductor confined in the elastomer. The *as*-prepared electrode is ultrastretchable to reach large uniaxial tensile strain up to 700%, as illustrated by the optical images in Figure 3(a). The exceptional deformability of the electrode is ascribed to the rapid transition into liquid-like behavior of yield-stress fluid upon tensile deformations, thereby resembling the functional liquids with the ultimate stretchability only limited by the fracture strain of the encapsulating elastomer [27]. The corresponding resistance shows a gradual increase in response to tensile deformations (Figure 3(b)). The normalized resistance follows the characteristic behavior of ideal conductive liquids expressed as (1 + *ε*)^2^, where *ε* is the uniaxial strain (see Figure S8). As the reversible solid/liquid transitions completely preserve the high conductivity of the yield-stress fluid, the resistance change is purely a geometric effect with the combined longitude extensions and cross-sectional contractions (see Figure 8 and S9). The yield-stress fluid electrode is further evaluated by strain-controlled fatigue tests. Figure 3(c) shows the evolution of the normalized resistance over 500 cycles of tensile deformations in the range from 100% to 400% strains. Notice that the samples are only allowed to relax at 100% strain due to a fairly long time is required for the full recovery of the viscoelastic encapsulation to the original state after large tensile deformations [15]. The resistance shows extremely stable responses in spite of repetitive mechanical strains, which demonstrates exceptional durability attractive for practical implementations. The inherent immunity to mechanical fatigue essentially resembles that of liquid state conductors [61].

**Figure 4:**
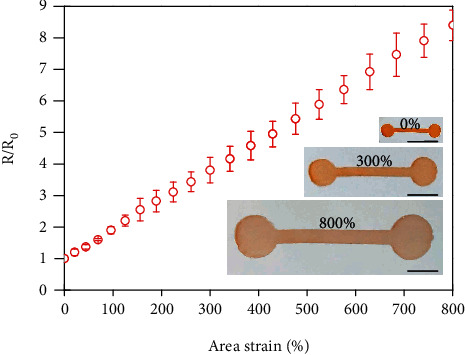
Yield-stress fluid electrode under biaxial tensile deformations. Normalized resistance as a function of area strain. Inset: optical images showing a yield-stress fluid electrode at 0%, 300%, and 800% area strains. Scale bar: 10 mm.

The yield-stress fluid electrodes are also compatible with large biaxial tensile deformations. In the inset of Figure 4, a series of optical images reveal the stable and uniform expansions of the electrodes. In Figure 4, the corresponding normalized resistance gradually increases with the area strain, which follows the predicted behavior of ideal liquids (see Figure S10 and S11). The transition into a liquid-state conductor results in well-expected resistance responses according to geometric deformations. The exceptional deformability of the yield-stress fluid electrode is essentially associated with the preservation of high ionic conductivity under various mechanical manipulations.

**Figure 5:**
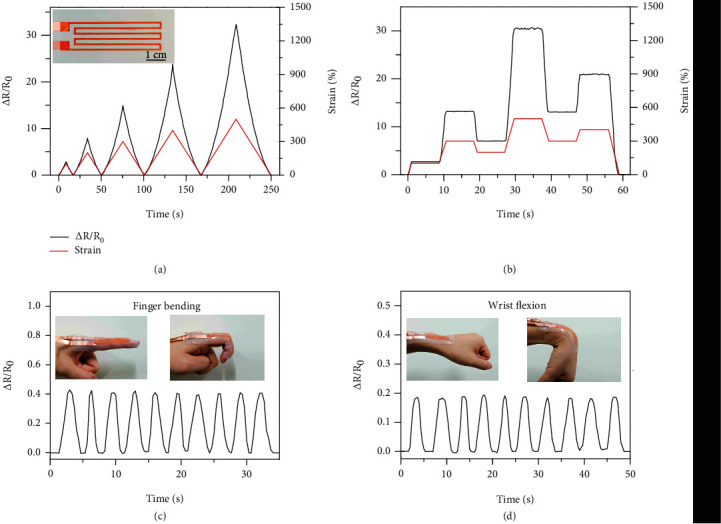
Strain sensors based on yield-stress fluid electrodes. (a) Normalized resistance change under cyclic uniaxial tensile deformations with the peak strains of 100%, 200%, 300%, 400%, and 500%. Inset: image of an *as*-prepared strain sensor. (b) Normalized resistance change in response to step deformations. (c) Real-time normalized resistance of the strain sensor to monitor dynamic motions of the index finger (c) and the wrist (d).

Highly stretchable yield-stress fluid electrode allows facile creation of transparent strain sensors for epidermal wearable electronics. The *as*-prepared transparent strain sensor is shown in Figure S12. In the inset of Figure 5, the electrode is colored in red for clear visualization of the patterning design consisting of interconnected parallel line segments. In Figure 5(a), the strain sensor was subjected to cyclic uniaxial tensile deformations with the peak strains progressively increased from 100% to 500%. The resistance is directly correlated with the applied strain and negligibly affected by the strain history. The normalized resistance and gauge factor are shown in Figure S13. In addition, the strain sensor exhibits fast and reliable responses to a series of step deformations, as shown in Figure 5(b). The durability is further demonstrated by the stable response curve after 1000 cycles of tensile deformations from 0 to 200% strains (see Figure S14). The strain sensor is readily attached to the skin by using silicone gel adhesive for monitoring the joint motions in real time, as shown in Figures 5(c) and 5(d). The maximum strains are estimated as 30% for finger bending and 16% for wrist flexion, respectively. The strain sensor demonstrates fast and reproducible responses to large tensile strains, which represents a promising wearable sensor to detect complex body movements.

**Figure 6:**
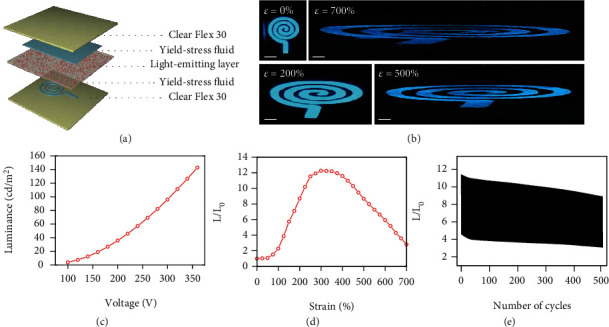
Stretchable alternating current electroluminescent (ACEL) device under uniaxial tensile deformations. (a) Schematic illustration of the ACEL device. (b) A series of optical images showing a representative device with a spiral-shaped luminous pattern at different uniaxial tensile strains. Scale bar: 5 mm. (c) Luminance versus voltage amplitude of a stretchable ACEL device powered by square-wave voltages at 25 kHz. (d) Normalized luminance as a function of tensile strain. (e) Evolution of normalized emission intensity over 500 stretch relaxation cycles in the strain range from 100% to 400%.

Compliant transparent electrodes also serve as key building components for emerging stretchable optoelectronics. As schematically illustrated in Figure 6(a), the yield-stress fluid electrodes are employed to prepare stretchable alternating current electroluminescent (ACEL) devices, in which the phosphor microparticles embedded in the electroluminescent layer are stimulated by alternative electric fields for light emissions. The luminous pattern is therefore defined by the overlapping area between the top and bottom transparent electrodes. The voltage-dependent emission characteristics are shown in Figure 6(c) for a representative ACEL device powered by a 25 kHz square-wave voltage. The luminance is 3.83 cd/m^2^ at 100 V, 35.76 cd/m^2^ at 200 V, and 111.2 cd/m^2^ at 320 V, respectively. Despite a thick electroluminescent layer design, the device can provide luminance of 100~200 cd/m^2^ at relatively low voltages typically required for indoor applications [62, 63], by using thermoplastic polyurethane as a polar dielectric matrix of the electroluminescent layer (see Figure S15). In addition, the luminance frequency characteristic with an applied voltage of amplitude 200 V is shown in Figure S16. The emission intensity initially increases with the frequency until 30 kHz as a result of increased injection of accelerated electrons to activate the luminance centers [64–66]. The luminance decreases by further raising the frequency due to the capture of injected electrons at donors [67–69].

**Figure 7:**
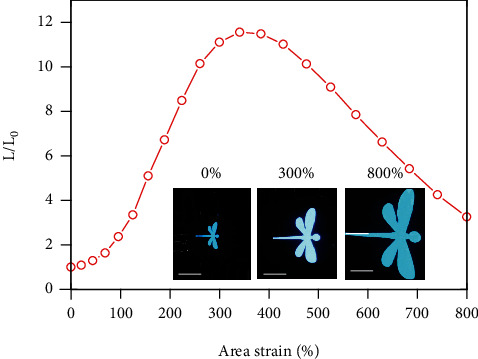
Stretchable ACEL device under biaxial tensile deformations. Normalized emission intensity as a function of area strain. Inset: optical images showing a dragonfly-shaped luminous pattern at 0%, 300%, and 800% area strains. Scale bar: 2 cm.

The deformability of the ACEL devices is evaluated by measuring their optoelectronic characteristics in response to various mechanical manipulations. In Figures 6(b), a series of optical images reveal a spiral-patterned device under uniaxial tensile deformations, which maintains stable and uniform light emission up to 700% strain. The dynamic deformation process of the ACEL device is further revealed in Movie S2. In Figure 6(d), the corresponding normalized luminance initially increases with tensile deformations until 300% strain, which is ascribed to enhanced electric fields by reducing the electroluminescent layer thickness. As the strain further increases, the light intensity continuously decreases due to the significant rise in resistance of the electrodes. Stretchable ACEL device is also assessed by repetitive uniaxial stretching in the strain range from 100% to 400%, as shown in Figure 6(e). The intensity is maintained at 78% of the initial value after 500 cycles, which verifies the excellent durability desirable for practical application settings. In addition to uniaxial stretching, the ACEL devices are capable of accommodating large biaxial tensile deformations. As illustrated by optical images in Figure 7, the device retains a stable and uniform dragonfly-shaped pattern up to 800% area strain. As the area expands, the corresponding emission intensity first increases and then decreases, with a peak value achieved at 340% area strain (see Figure 7). The trend also comes from the competition between the enhanced electric field and the increased electrode resistance.

## 3. Discussions

In summary, we have developed a yield-stress fluid by dispersing silica nanoparticles into a concentrated aqueous electrolyte, which functions as viscous ink for screen printing of highly conductive and ultrastretchable transparent conductors over various elastomer substrates. The printed features are solid state in nature with exceptional structural retention as a result of the three-dimensional colloid network. The transition into liquid-like behavior upon tensile deformation results in ultrahigh stretchability up to the fracture strain of the elastomer substrates. The practical suitability is demonstrated by wearable strain sensors and stretchable light-emitting devices. The yield-stress fluids allow facile and scalable fabrication of high-performing transparent conductors, which may find a broad range of applications in stretchable optoelectronic devices and systems.

## 4. Materials and Methods

### 4.1. Materials and Preparations

All chemical reagents were commercially acquired including polyethylene oxide (PEO, *M*_*w*_ = 300 k) from Shanghai Aladdin Bio-Chem Technology Co., Ltd., lithium chloride (LiCl) and sodium chloride (NaCl) from Shanghai Energy Chemical Co., Ltd., and fumed silica (AEROSIL 380) from Evonik Degussa GmbH. Thermoplastic elastomers were purchased from corresponding vendors including thermoplastic polyurethane (TPU, Estane T460A) from Lubrizol Inc., styrene−isoprene-styrene (SIS, D1113) from Kraton Corporation, and PVDF-HFP (Daiel G801) from Daikin Industries. Silicone (Ecoflex 00-30 A/B) and polyurethane (PU, Clear Flex 30 A/B) were obtained from Smooth-On, Inc. Cu-doped ZnS phosphor microparticles were synthesized by Shanghai Keyan Phosphor Technology Co., Ltd. The VHB 4910 tape of 1 mm in thickness was obtained from 3 M Inc. The ionically conductive liquid was prepared by dissolving high concentrations of selected salts in an aqueous PEO solution (5 w/v %), in terms of 4 mol/L for NaCl and 10 mol/L for LiCl, which approached the solubility under the ambient conditions [60]. The yield-stress fluids were formed by further addition of fumed silica (10 w/v %) as a rheological modifier, followed by homogenization in a planetary ball mill (YXQM, Changsha MITR Co., Ltd.) for 2 h. As regards the preparation of stretchable substrates, all thermoplastic elastomers were dissolved in selected solvents and then drop cast onto nonsticky glass wafers functionalized with octadecyltrichlorosilane (OTS), followed by natural evaporation to thoroughly remove the solvents. The solvent choices and concentrations were adjusted to achieve optimal quality of the resulting substrates, including toluene for tetrahydrofuran for TPU (20 w/v %), SIS (20 w/v %), and 4-Methyl-2-pentanone for PVDF-HFP (25 w/v %). Silicone substrates were obtained by drop-casting mixed precursor of Ecoflex 00-30 A/B onto OTS-modified glass wafers and then thermally curing at 120°C for 2 h. All printing processes were carried out on a manual screen printer (Zhuhai Kaivo Electronic Co., Ltd.) by using stainless steel stencil masks.

### 4.2. Material Characterizations

Structural characterizations were carried out by optical microscopy and confocal laser scanning microscopy by using a Keyence VK-X1000 microscope. Optical images were captured using a Fujifilm X-T10 digital camera. Rheology experiments were conducted on a HAAKE RheoStress 600 instrument. Steady and dynamic oscillatory shear measurements were conducted at room temperature by using a set of 35 mm diameter parallel plates with a sample thickness of 0.5 mm. The frequency was fixed at 1 Hz in all the dynamic oscillatory shear measurements. The optical transmittance spectra were measured by a fiber optical spectrometer (Ideaoptics PG2000-Pro) equipped with a 6 cm diameter integrating sphere. The impedance of ionic conductors was measured using a GW Instek LCR Meter (Model LCR-6300). The storage stability was evaluated in an environmental chamber at 23°C and 20% relative humidity. The tensile deformations were applied by homemade motorized translation stages. The dielectric constants of elastomers were measured with parallel plate capacitor structures by the LCR Meter. The frequency-dependent dielectric constant of TPU is shown in Figure S15.

### 4.3. Strain Sensor Fabrication and Evaluation

A 100 *μ*m-thick silicone substrate was prepared on an OTS-modified glass wafer. The sensor electrode of ~150 *μ*m in thickness was defined by stencil printing, followed by drop-casting a layer of Ecoflex 00-30 for encapsulation. The *as*-prepared strain sensor was brush painted with a layer of silicone gel (Silbione RT GEL 4645A/B, Elkem Silicones) as the biocompatible adhesive for skin attachment. The resistance of the strain sensor was measured by the LCR meter. The uniaxial tensile strains were applied by a homemade motorized translation stage. The step deformations were applied at the maximal translation speed of the stage (40 mm/s).

### 4.4. Light-Emitting Device Fabrication and Evaluation

A 100 *μ*m-thick electroluminescent layer was prepared by doctor-blade coating with dissolved electroluminescent composite over OTS-modified glass. Spin cast TPU dielectric layers of 15 *μ*m in thickness were thermally laminated onto both sides of the electroluminescent layer to improve the dielectric strength. Subsequently, ~150 *μ*m-thick conductive electrode was generated by stencil printing and encapsulated by drop-cast PU precursor (Clear Flex 30 A/B). After curing under the ambient condition for 48 h, the VHB tape was laminated on top as the substrate. The entire structure was peeled off from the glass wafer, flipped over, and attached to an OTS-modified glass wafer. The aforementioned printing and encapsulation steps were repeated to prepare the other transparent electrode. A high-voltage amplifier (Trek, Model 10/10B-HS) was employed to power the ACEL devices with well-defined waveforms supplied by a GW Instek AFG-2500 function generator. Emissive properties were quantified using a luminance and color meter (Konica Minolta CS-150).

## Data Availability

All data needed to evaluate the conclusions in the paper are present in the paper and/or the Supplementary Materials. Additional data related to this paper may be requested from the authors.

## Supplementary Materials

Supplementary 1 Figure S1: long-term stability of yield-stress fluids. Figure S2: influence of fumed silica concentration on the rheological properties. Figure S3: influence of PEO and silica additives on the rheological properties. Figure S4: influence of fumed silica concentration on the printing quality. Figure S5: influence of PEO concentration on the printing quality. Figure S6: conductivity of yield-stress fluid as a function of PEO concentration. Figure S7: influence of LiCl concentration on water retention. Figure S8: theoretical and experimental electromechanical properties of yield-stress fluid electrode under uniaxial tensile deformations. Figure S9: electromechanical properties of yield-stress fluid electrodes of different thicknesses under uniaxial tensile deformations. Figure 10: theoretical and experimental electromechanical properties of yield-stress fluid electrode under biaxial tensile deformations. Figure S11: electromechanical properties of yield-stress fluid electrodes of different thicknesses under biaxial tensile deformations. Figure S12: optical images of an *as*-printed transparent strain sensor. Figure S13: gauge factor of the strain sensor. Figure S14: durability of the strain sensor. Figure S15: dielectric constant of TPU elastomer. Figure S16: frequency-dependent luminance of a representative ACEL device.

Supplementary 2 Movie S1. A yield-stress fluid under static and shaking conditions.

Supplementary 3 Movie S2. A stretchable ACEL device retaining a stable luminous pattern under uniaxial deformation in the strain range from 0 to 700%.
