# Supplementary material for: A Printable and Conductive Yield-Stress Fluid as an Ultrastretchable Transparent Conductor
Source: Research (Wash D C). 2021 Dec 14;2021:9874939. doi: 10.34133/2021/9874939 (PMC8696283; doi:10.34133/2021/9874939)
Supplement: Supplementary 1 — Figure S1: long-term stability of yield-stress fluids. Figure S2: influence of fumed silica concentration on the rheological properties. Figure S3: influence of PEO and silica additives on the rheological properties. Figure S4: influence of fumed silica concentration on the printing quality. Figure S5: influence of PEO concentration on the printing quality. Figure S6: conductivity of yield-stress fluid as a function of PEO concentration. Figure S7: influence of LiCl concentration on water retention. Figure S8: theoretical and experimental electromechanical properties of yield-stress fluid electrode under uniaxial tensile deformations. Figure S9: electromechanical properties of yield-stress fluid electrodes of different thicknesses under uniaxial tensile deformations. Figure 10: theoretical and experimental electromechanical properties of yield-stress fluid electrode under biaxial tensile deformations. Figure S11: electromechanical properties of yield-stress fluid electrodes of different thicknesses under biaxial tensile deformations. Figure S12: optical images of an as-printed transparent strain sensor. Figure S13: gauge factor of the strain sensor. Figure S14: durability of the strain sensor. Figure S15: dielectric constant of TPU elastomer. Figure S16: frequency-dependent luminance of a representative ACEL device. [file 9874939.f1.docx]

Supplementary Information

**A Printable and Conductive Yield-stress Fluid as An Ultrastretchable Transparent Conductor**

Qianying Lu^1^, Yunlei Zhou^1^, Xiangfei Yin^2^, Shitai Cao^1^, Xiaoliang Wang^2^*, and Desheng Kong^1^*

^1^ College of Engineering and Applied Sciences, State Key Laboratory of Analytical Chemistry for Life Science, and Jiangsu Key Laboratory of Artificial Functional Materials, Nanjing University, Nanjing 210046, China.

^2^ Key Laboratory of High Performance Polymer Materials and Technology of Ministry of Education, Department of Polymer Science and Engineering, School of Chemistry and Chemical Engineering, Nanjing University, Nanjing 210046, China.

* Correspondence should be addressed to Desheng Kong; dskong@nju.edu.cn and Xiaoliang Wang; wangxiaoliang@nju.edu.cn

Supplementary Figures


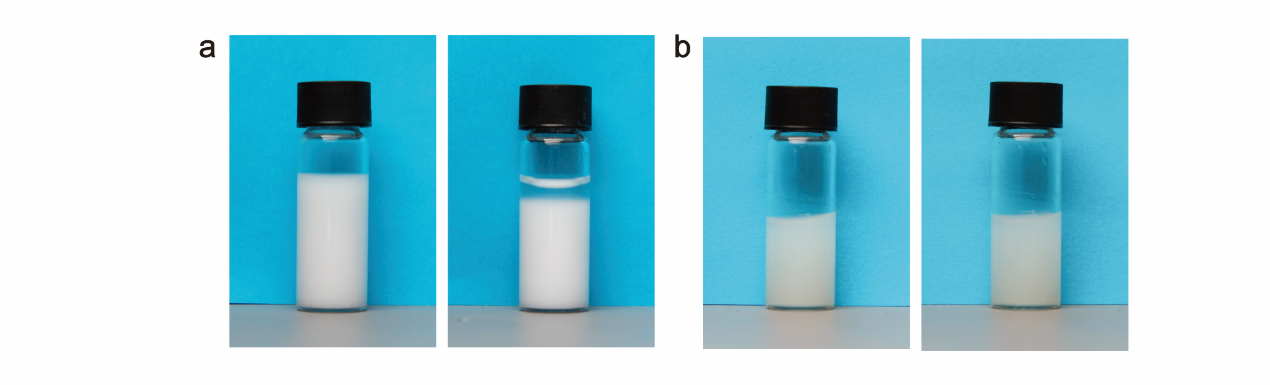


**Figure S1.** Long-term stability of yield-stress fluids. (a) Optical images of an aqueous SiO_2_ dispersion (10 w/v %) at the original state (left) and after 30 days (right). The obvious sedimentation of the dispersion suggests the practical issue of long-term stability. (b) Optical images of a mixed aqueous dispersion of SiO_2_ (10 w/v %) and PEO (5 w/v %) at the original state (left) and after 30 days (right).


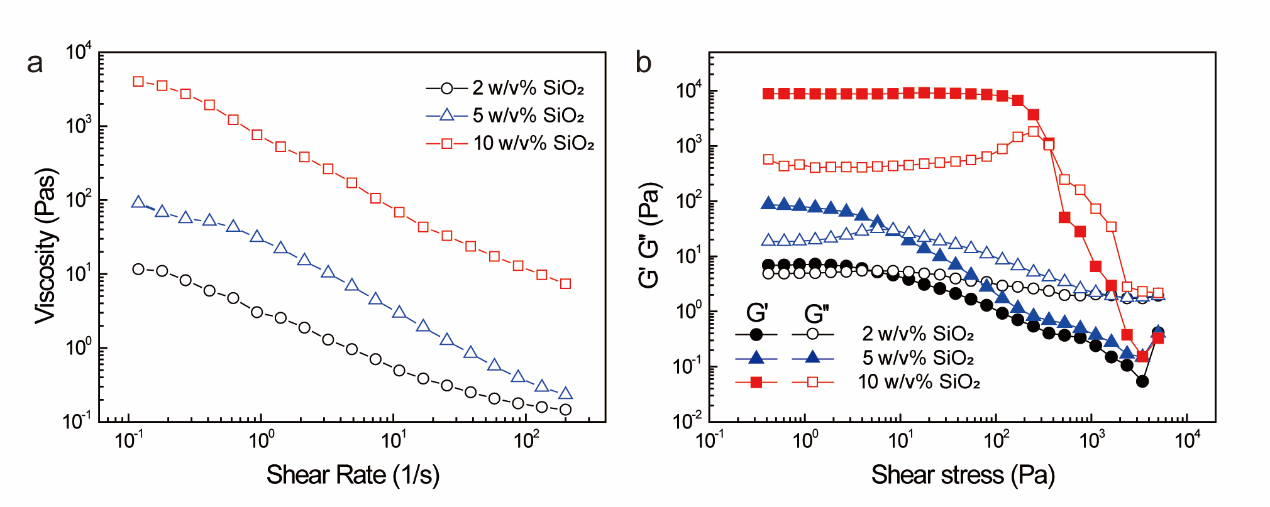


**Figure S2.** Influence of fumed silica concentration on the rheological properties. (a) Viscosity versus shear rate for yield-stress fluid formulations containing different concentrations of SiO_2_. The viscosity largely increases with the addition of SiO_2_ across the entire frequency range. (b) Storage moduli (G′) and loss moduli (G″) as a function of shear stress. The additional SiO_2_ effectively boosts the quasi-static storage moduli and the shear yield stress.


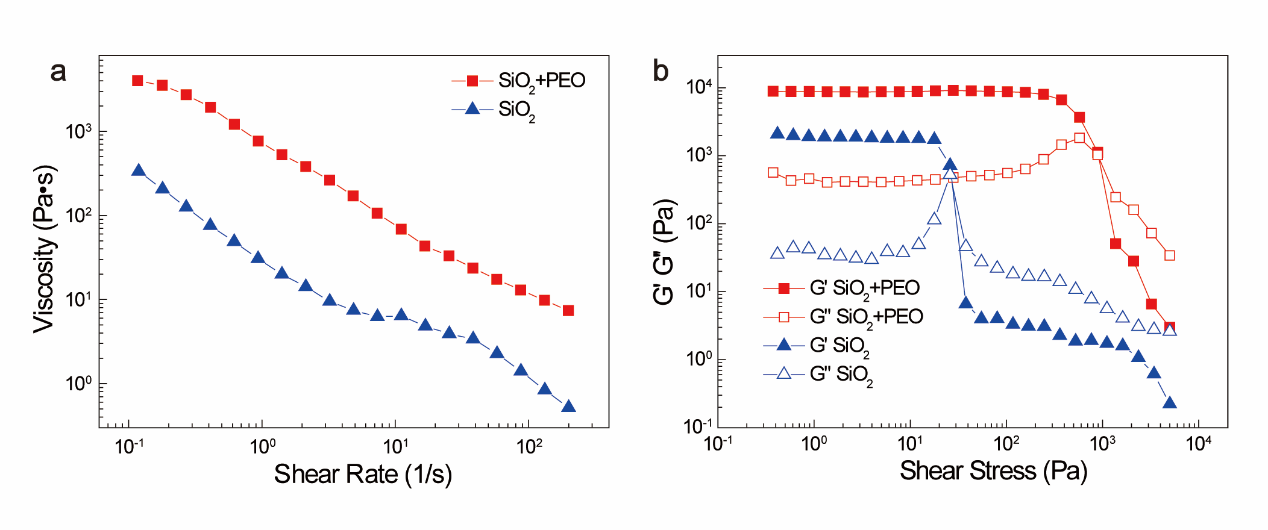


**Figure S3.** Influence of PEO and silica additives on the rheological properties. (a) Viscosity versus shear rate for yield-stress fluid formulations containing rheological modifiers of SiO_2_ and SiO_2_/PEO, which exhibit pronounced shear thinning behaviors. The addition of PEO notably increases the viscosity across the entire frequency range. (b) Storage moduli (G′) and loss moduli (G″) as a function of shear stress. The further addition of PEO effectively enhances quasi-static G' and the shear yield stress.


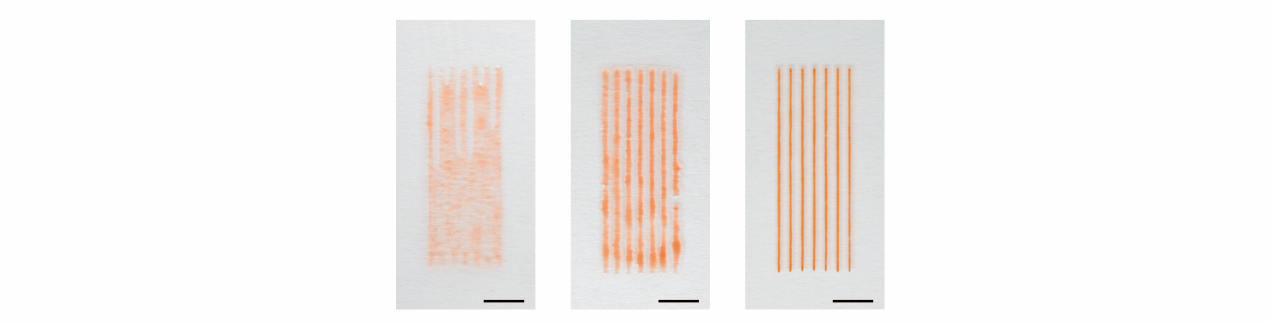


**Figure S4.** Optical images of printed stripe pattern based on yield-stress fluids containing 2 (left), 5 (middle), and 10 (right) w/v% SiO_2_. All formulations also consist of 10 mol/L LiCl and 5 w/v% PEO. Scale bar: 5mm.


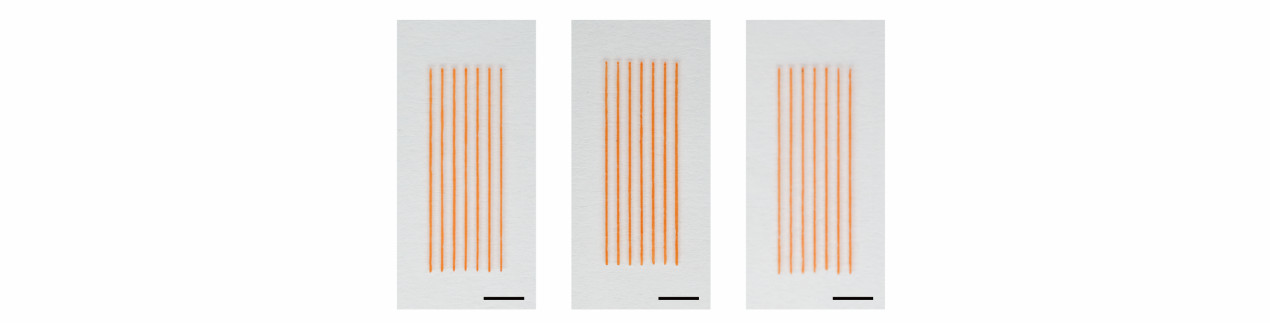


**Figure S5.** Optical images of printed stripe pattern based on yield-stress fluids containing 5 (left), 7.5 (middle), and 10 (right) w/v% PEO. All formulations also consist of 10 mol/L LiCl and 10 w/v% SiO_2_. Scale bar: 5mm.


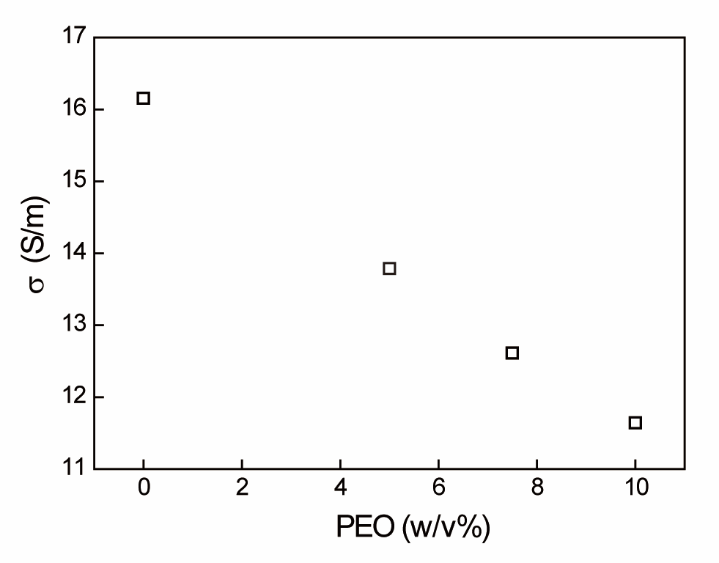


**Figure S6.** Conductivity of yield-stress fluids as a function of PEO concentration. All formulations also consist of 10 mol/L LiCl and 10 w/v% SiO_2_. The conductivity continuously declines by increasing PEO concentration.


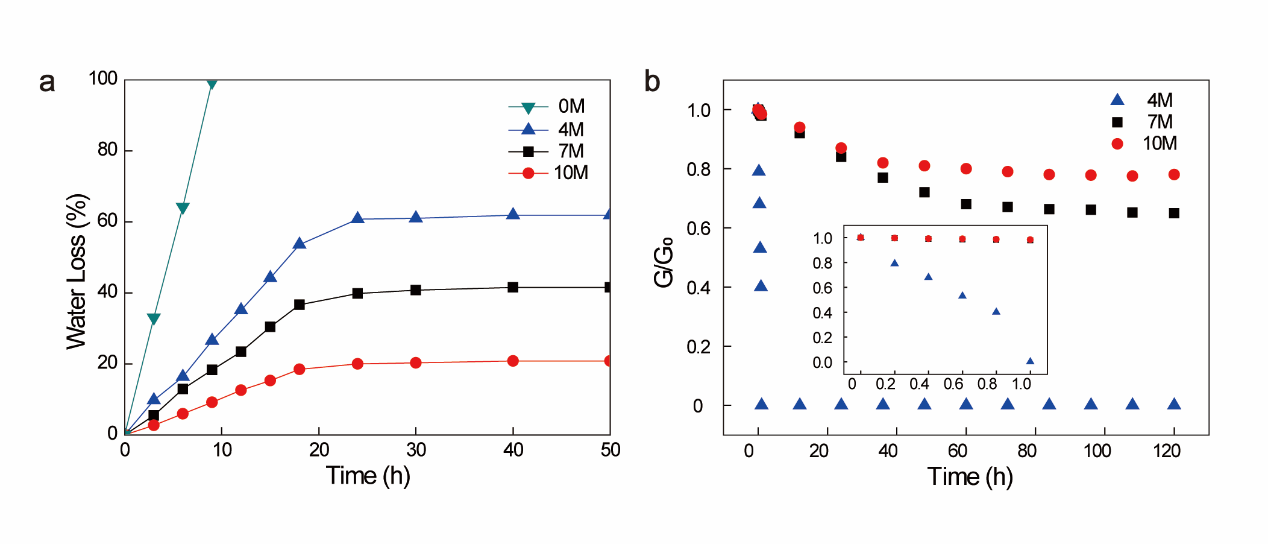


**Figure S7.** (a) Water loss as a function of time of yield-stress fluids containing different concentrations of LiCl stored in an environmental chamber at 23 ℃ and 20% relative humidity. The yield-stress fluid based on deionized water completely dries out within 9 h. After the addition of LiCl salt, the water loss shows gradual increase and then approaches to the steady-state value. The water retention is improved by increasing with LiCl concentration. (b) Conductivity versus storage time. The yield-stress fluid based on 4 M LiCl shows rapid loss of conductivity within 1 h due to insufficient water retention. The conductivity values of yield-stress fluids with high LiCl concentrations of 7 M are 10 M are largely preserved to enable long-term storage.


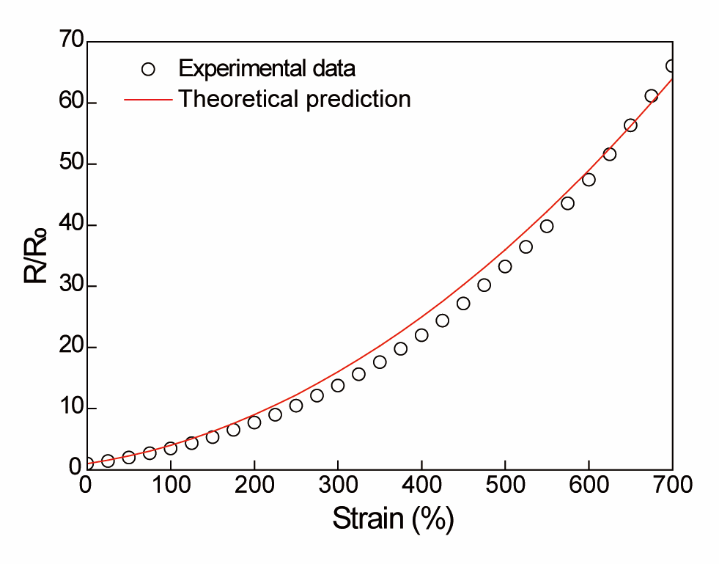


**Figure S8.** Normalized resistance as a function of uniaxial tensile strain for a yield-stress fluid electrode and an ideal liquid-state conductor. The electrode thickness is 150 μm. Assuming the length is L0 and the cross-section area is A_0_ at the relaxed state, the resistance of the ideal liquid-state conductor is described by the expression $R_{0}=L_{0}/(A_{0} )$, in which σ is the conductivity. At uniaxial tensile strain ε, the length is expressed as L = (ε+1)L_0_. The ideal liquid state conductor is considered as incompressible, so the cross-section area follows A = A_0_/(ε+1). Accordingly, the resistance under tensile deformation is described by $R=L/A$ = (ε+1)^2^ L_0_/A_0_ = (ε+1)^2^R_0_.


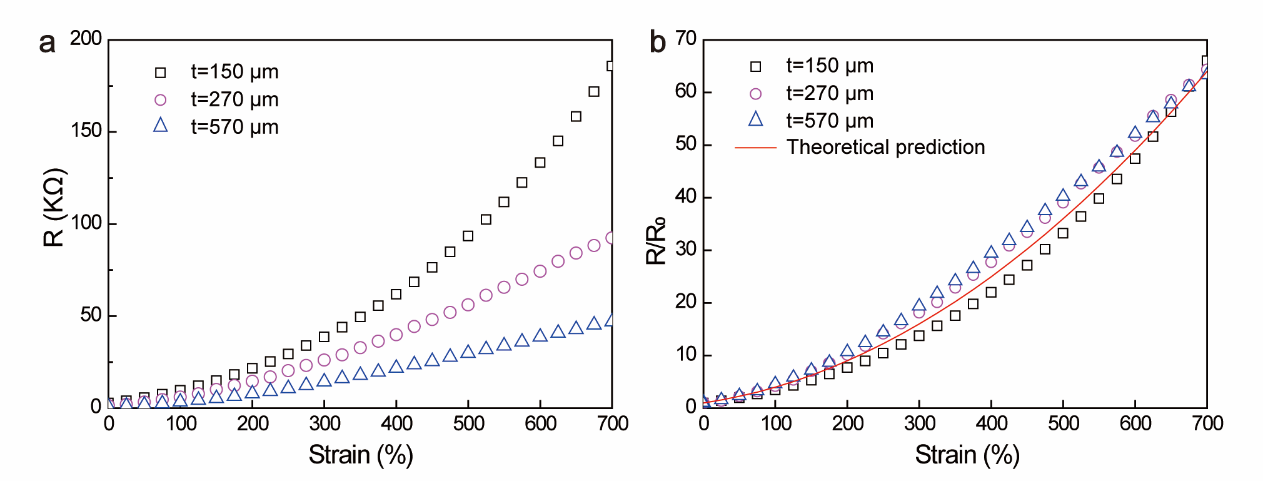


**Figure S9.** (a) Resistance *versus* uniaxial tensile strain for yield-stress fluid electrodes with of three different thicknesses. (b) Normalized resistance *versus* uniaxial tensile strain. The sheet resistance of the electrode is 400 Ω/sq. at 150 μm, 220 Ω/sq. with at 270 μm, and 105 Ω/sq. at 570 μm, respectively thickness. The electromechanical properties of the electrodes essentially follow the predicted behavior of the ideal liquid.


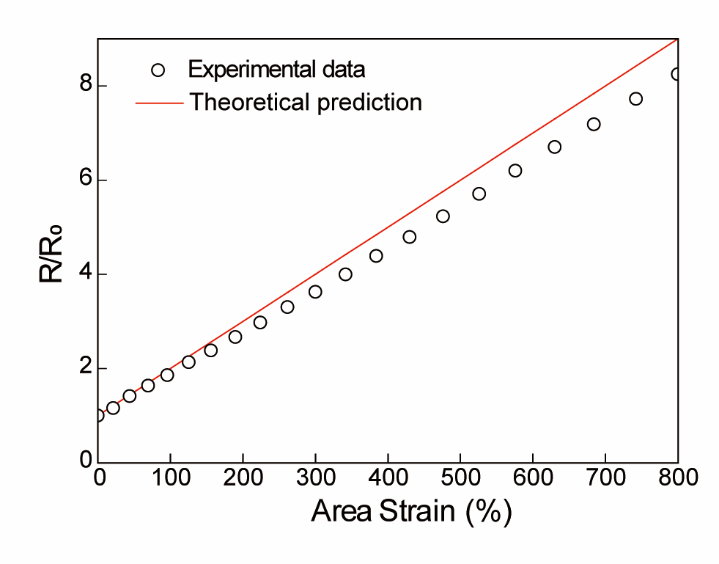


**Figure S10.** Normalized resistance as a function of biaxial tensile strain for a yield-stress fluid electrode and an ideal liquid-state conductor. The electrode thickness is 150 μm. Assuming the length is L_0_ and the cross-section area is A_0_ at the relaxed state, the resistance of the ideal liquid-state conductor is described by the expression $R_{0}=L_{0}/(A_{0} )$, in which σ is the conductivity$.$ At biaxial tensile deformation with an area strain εA_0_, the length is expressed as L = $\sqrt{\varepsilon+1}$L_0_. The ideal liquid state conductor is considered as incompressible, so the cross-section area follows A = A_0_/$\sqrt{\varepsilon+1}$. Accordingly, the resistance under tensile deformation is described by $R=L/A$ = (ε+1) L_0_/A_0_ = (ε+1)R_0_.


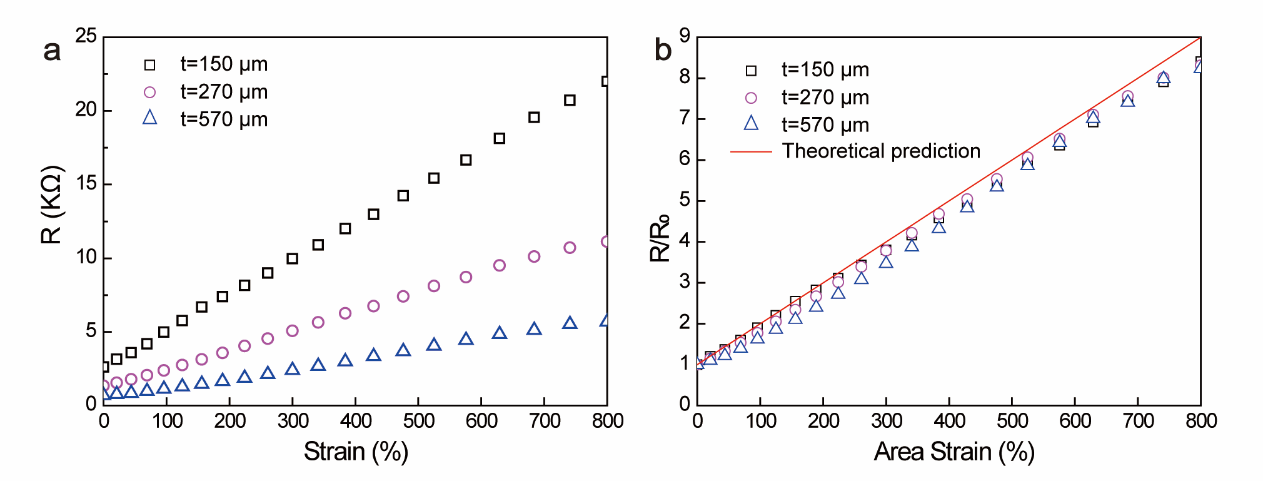


**Figure S11.** (a) Resistance *versus* biaxial tensile strain for yield-stress fluid electrodes of different thicknesses. (b) Normalized resistance *versus* biaxial tensile strain. The sheet resistance of the electrode is 400 Ω/sq. at 150 μm, 220 Ω/sq. with at 270 μm, and 105 Ω/sq. at 570 μm, respectively thickness. The electromechanical properties of the electrodes essentially follow the predicted behavior of ideal liquids.


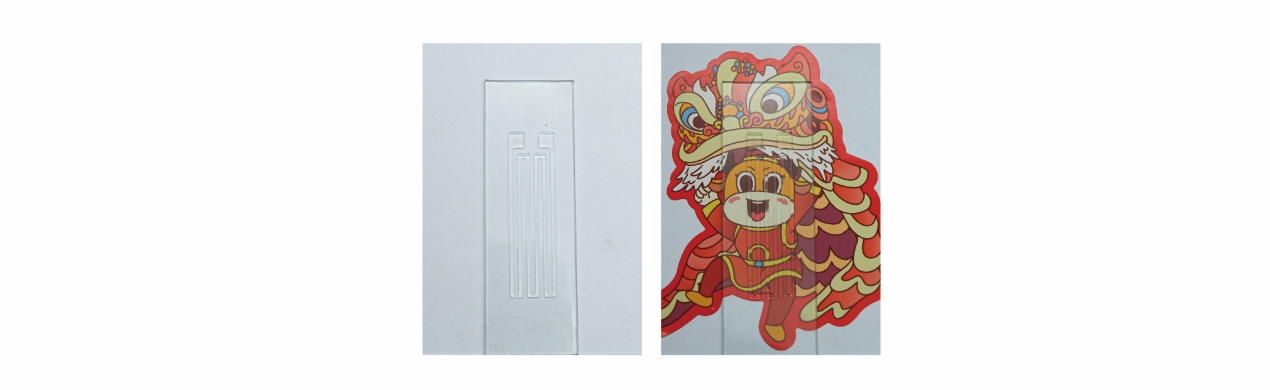


**Figure S12.** Optical images of printed transparent strain sensor in white (left) and colorful (right) background.


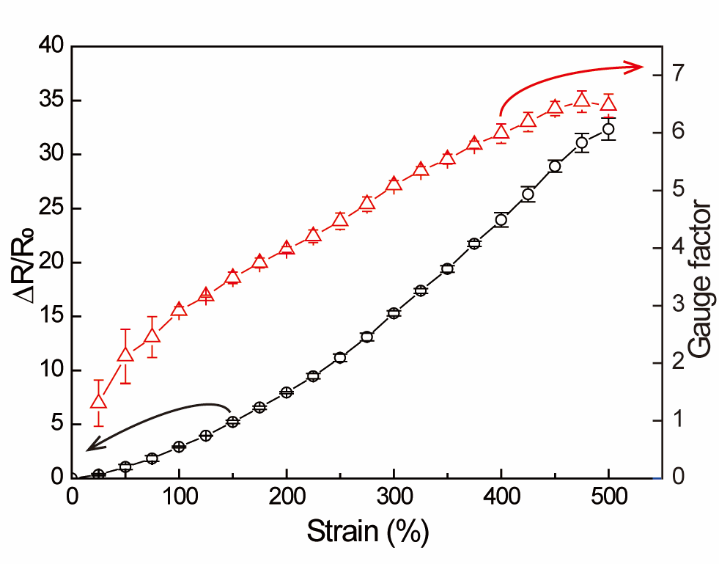


**Figure S13.** Normalized resistance change and gauge factor (GF) of the strain sensor as a function of tensile strain.


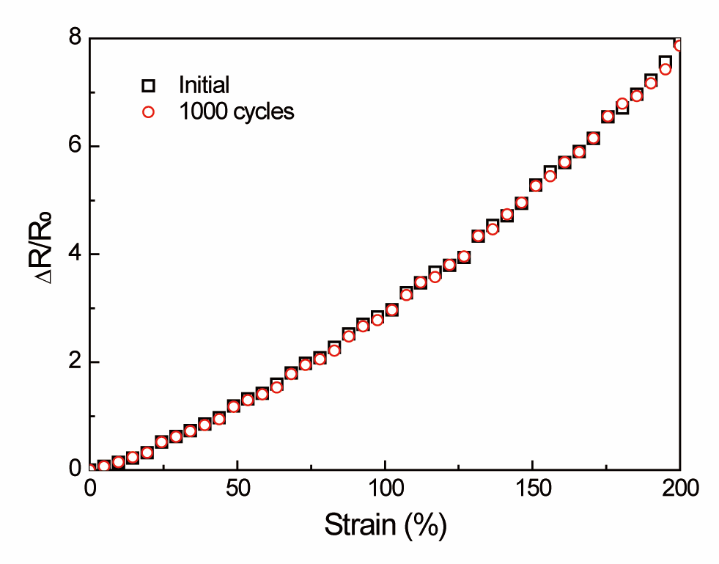


**Figure S14.** Normalized resistance change of the strain sensor as a function of strain before and after tensile fatigue test. The tensile fatigue test involves 1000 stretch-relaxation cycles from 0 to 200% strains.

**
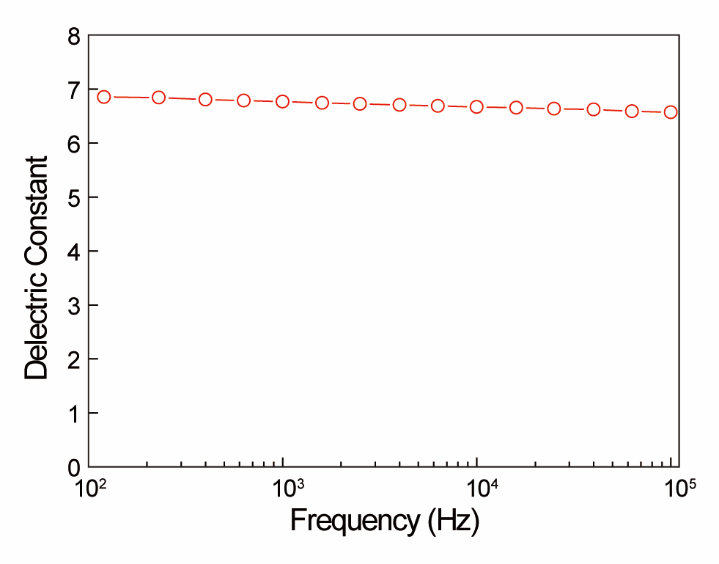
**

**Figure S15**. Dielectric constant of TPU in the frequency range from 10^2^ to 10^5^ Hz. The polar elastomer exhibits a high dielectric constant of 6.77 at 1 kHz.


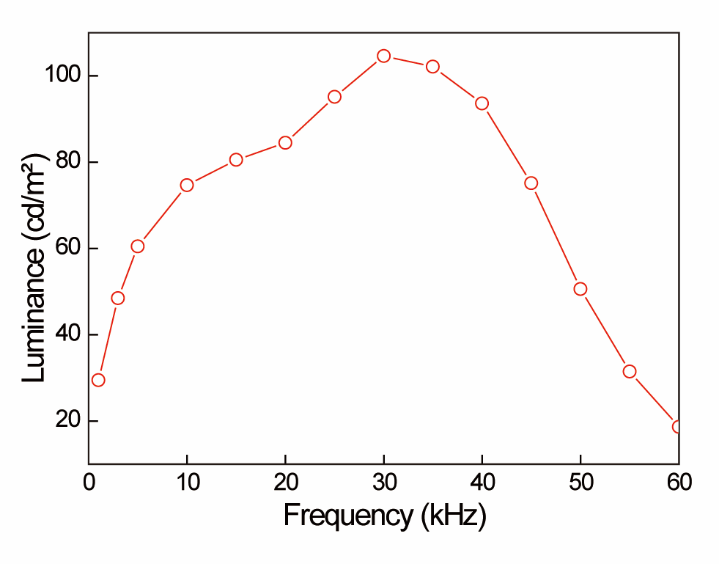


**Figure S16**. Frequency-dependent luminance of a representative ACEL device powered by 300 V square wave voltages. The maximal emission intensity is obtained at an intermediate frequency of 30 kHz.

Supplementary Movies

**Movie S1.** A yield-stress fluid under static and shaking conditions.

**Movie S2.** A stretchable ACEL device retaining a stable luminous pattern under uniaxial deformation in the strain range from 0 to 700%.
